# Intimate Partner Homicides in Norway 1990–2020: An Analysis of Incidence and Characteristics

**DOI:** 10.1177/08862605211063508

**Published:** 2022-01-04

**Authors:** Solveig K. B. Vatnar, Christine Friestad, Stål Bjørkly

**Affiliations:** 1 Centre for Research and Education in Forensic Psychiatry, 155272 Oslo University Hospital, Oslo, Norway; 2 Faculty of Health and Social Sciences, 5562 Molde University College, Oslo, Norway; 3 University College of Norwegian Correctional Service, Oslo, Norway

**Keywords:** intimate partner homicide, intimate partner violence, time changes, incidence rates, risk factor

## Abstract

Intimate partner homicide (IPH) is an extreme outcome of intimate partner violence (IPV). It is a societal challenge that needs to be investigated over time to see whether changes occur concerning the incidence of IPH, IPH characteristics, socioeconomic factors, and contact with service providers. This study includes the total Norwegian cohort of IPHs between 1990 and 2019 with a final conviction (*N* = 224). Poisson regression was applied to model the incidence rate of homicide and IPH between 1990 and 2020 as well as the incidence rates of immigrant perpetrators and victims. Multivariate logistic regression analyses were used to test the association between characteristics and period 1990–2012 compared to after 2012 as dependent variable. The results show that though homicide incidence rates in Norway declined steadily and significantly after 1990, IPH rates did not begin to decline until 2015. The following IPH characteristics showed reduced incidence after 2012: IPH-suicide, perpetrators with a criminal record, and IPHs perpetrated subsequent to preventive interventions towards the perpetrator. Sentence length in IPH cases had increased. Changes were not observed for any of the other IPH characteristics investigated. IPH is often the culmination of long-term violence and can be prevented, even if risk assessment is challenging due to the low base rates.

## Background

Intimate partner violence (IPV) is a health, social, and justice issue, but also a matter of human rights and citizenships (e.g., Matias et al., 2020; Spencer & Stith, 2018; UNODC, 2019; World Health Organization, 2014). While historically conceived as an individual problem in the private domain, IPV is increasingly recognized as a serious crime and a social problem with complex implications (e.g., Dobash & Dobash, 2015; UNODC, 2019). Intimate partner homicide (IPH) represents an extreme outcome of IPV (Aldridge & Browne, 2003; Campbell et al., 2007; Campbell & Glass., 2009; Dobash et al., 2009; Matias et al., 2020; Nicolaidis et al., 2003; Spencer & Stith, 2018; Vatnar et al., 2017a). Since 2000, the number of deaths by interpersonal homicide has exceeded the number of war- and terrorism-related deaths (Matias et al., 2020; UNODC, 2019; WHO, 2014). Although women account for a far smaller proportion of total homicide victims than men, they bear, by far, the greatest burden of IPH (Stöckl, et al., 2013; UNODC, 2019). In order to address this reality, several countries have undertaken different actions to address IPV and IPH by adopting legal changes, early interventions, and multi-agency efforts, as well as by creating special units and implementing training in the criminal justice system (UNODC, 2019). To what extent, then, is the increased awareness of intimate partner violence as a serious social problem paralleled in changes over time in the empirical landscape of IPH? A recurring question from researchers, service providers, and policy makers has been whether substantial changes have occurred in more recent times concerning incidence of IPH, IPH characteristics, socioeconomic factors, and contact with service providers before an IPH.

### Incidence Rates for IPH

Globally, the prevalence of intimate partner/family-related homicide is increasing (UNODC, 2019). In 2017, more than half (58%) of female homicide victims were killed by intimate partners or other family members, while the prevalence was 47% in 2012 (UNODC, 2019). The regions with the largest *number* of females killed by IPH were Asia (20,000) and Africa (19,000), followed by the Americas (8000), Europe (3000), and Oceania (300) (UNODC, 2019). As population sizes differ widely between regions, prevalence is better represented by rates. The highest *rates* were in Africa (3.1 per 100,000 female population), followed by the Americas (1.6), Oceania (1.3), Asia (0.9), and Europe (0.7). Therefore, even though the largest number of women and girls are killed by intimate partners or other family members in Asia, women’s risk of being killed by an intimate partner or another family member is highest in Africa (UNODC, 2019).

Globally, the largest proportion of women killed in IPH is seen in Oceania (42%), while Europe accounts for the lowest proportion (29%) (UNODC, 2019). However, there may be considerable within-region differences, as illustrated by findings from Norway where 48% of women killed in the time span 2011–2020 were victims of IPH (Kripos/NCIS, 2021).

Research on time changes within IPV and IPH has primarily focused on long-term changes in femicide (Powers & Kaukinen, 2012). When investigating changes in IPV from 1980 to 2008 in the U.S., women’s employment was associated with an increase in women’s risk of IPV, partly contingent on the victim’s race (Powers & Kaukinen, 2012).

Generally, homicide rates increased in the 1960s, followed by a steady decline from early 1990s in the U.S. and Western Europe (e.g., Aebi & Linde, 2014). Some studies have found a similar pattern for IPHs in the U.S., Canada, and Western Europe (e.g., Caman et al., 2017; Corradi & Stöckl, 2014; Dawson et al., 2009). Globally, however, the prevalence of intimate partner/family-related homicide is increasing (UNODC, 2019).

Western European countries have the lowest homicide rates worldwide (UNODC, 2019). In 2010, it was suggested that a substantial decline in homicide rates was unrealistic and that a minimum threshold for homicides might have been reached (Aebi & Linde, 2014). Only a few studies have investigated time changes in IPH specifically (Caman, et al., 2017; Corradi & Stöckl, 2014; Spencer & Stith, 2018; UNODC, 2019). Studies from the U.S. reported the largest decline being in female perpetrators with male victims (e.g., Corradi & Stöckl, 2014; Fox & Zawitz, 2007). This showed a corresponding increase in the proportion of IPH with female victims between 1976 and 2005 (Fox & Zawitz, 2007). In contrast, findings from Sweden showed a decrease in male-perpetrated IPH and stable trends for female-perpetrated IPHs from 1990 to 2013 (Caman et al., 2017). Findings from Canada indicated an overall decline in IPH from 1976 to 2001, with a stronger reduction in IPHs with male than female victims after 1991 (Dawson et al., 2009). Findings from Norway indicated stable IPH incidence between 1990 and 2012 (Vatnar et al., 2017a). Thus far, recent findings indicate that even in regions with decreasing homicide rates, the decline of IPH is modest in comparison to other types of homicides and actually appears to be rather constant over time (UNODC, 2019).

### Characteristics of IPH

According to the United Nations, 8 out of 10 IPH victims are female, and this disproportion has remained quite stable (UNODC, 2019). When studying time changes and gender as a characteristic of the IPH perpetrator, the results are mixed and inconclusive. The U.S. and Canadian studies have reported a decline primarily of female perpetrators, and a Swedish study found a modest fall in male-perpetrated IPHs and rates of female-perpetrated IPHs being stable (Caman et al., 2017; Dawson et al., 2009; Fox & Zawitz, 2007; Reckdenwald & Parker, 2012). A U.S. study identified the largest decline in Black male victims of IPH, while the proportion of female victims increased from 1976 to 2005 (Fox & Zawitz, 2007). Findings reported from Canada for 1991 to 2001 showed a decrease of 4.4 to 2.0 per million for male victims and 16.5 to 8.0 for female victims (Dawson et al., 2009).

The existing literature examining female compared to male perpetrators is scarce, restricted to two single studies. Dawson et al. (2009) findings from Canada indicated that shifts in relative employment and divorce rates were associated with declining rates for male-perpetrated IPH. Changing divorce rates were also associated with lowered rates of female-perpetrated IPH, as were changes in men’s education. The Caman and colleagues’ (2017) study from Sweden indicated a shift in male-perpetrated IPH cases; they were less likely to involve previous IPV, alcohol intoxication at the time of offense, and were less likely to be followed by suicide. Similar changing characteristics were not observed for female-perpetrated IPHs. Still, two recent meta-analyses have supported previous IPV as the strongest risk factor for IPH (Matias et al., 2020; Spencer & Stith, 2018). Further, perpetrator-related risk factors were Black ethnicity, lower education level, suicidal thoughts and attempts, prior criminal records, and a history of IPV in a previous relationship (Matias et al., 2020). Victim-related risk factors were Black ethnicity, foreign nationality, lower education level, and alcohol consumption (Matias et al., 2020). Among precipitating and situational characteristics of IPH, the victim and the perpetrator being under the influence of alcohol stood out, as did access to firearms and other weapons/sharp objects, and the context of victims’ or shared residence (Matias et al., 2020). Jealousy and disputes are considered motive-related risk factors for IPH (Matias et al., 2020; Spencer & Stith, 2018; Vatnar et al., 2017a), while relationship characteristics such as length of relationship, marital status, or having children have not been supported as significant risk factors for IPH (Spencer & Stith, 2018).

Ethnic minority status is considered an important risk factor for IPH (Matias et al., 2020), although it may be that social and economic disadvantages, rather than ethnicity per se, are the actual, underlying issues (Barrett & St Pierre, 2011; Dobash et al., 2009; Vatnar et al., 2017b). Thus, ethnic or other socioeconomic minority status indicators must be considered when addressing changes of IPH across time.

### Contact with Service Providers Previous to the IPH

Previous proposals suggested that men’s homicidal behavior against women remains the same regardless of the availability of resources to battered women (Dugan et al., 2003). Resources—like shelters—have even been associated with a decline in *male* IPH victims (Browne et al., 1999). One interpretation of this finding is that increased opportunities for leaving a violent partner prevented IPH being committed in self-defense. Thus, actions aimed at protecting women from IPH victimization resulted in protecting IPH male victims (Dugan et al., 2003). It has been speculated that health service quality is less relevant for IPHs than other categories of homicide, as IPHs tend to take place inside a home in the absence of witnesses and are characterized by excessive violence (e.g., Caman et al., 2017; Dutton, 2002). However, recent findings indicate that a substantial proportion of IPH perpetrators and victims were known to the authorities and service providers before the IPH (UNODC, 2019; Vatnar et al., 2017a).

### Explanatory Framework

Recent studies highlight the apparent heterogeneity of IPV, including variability in IPV types (e.g., physical, sexual, and psychological), severity (e.g., minor, major, and lethal), function (e.g., threats, situational, and continuous), and victim/perpetrator roles (e.g., mutual and intimate terrorism) (e.g., Bell & Naugle, 2008; Winstok, 2007). A multi-disciplinary interactional perspective, which takes into consideration the characteristics, perspectives, and interplay of perpetrators, victims, and context has been proposed to create a more comprehensive theoretical approach. An interactional perspective on IPV may increase theoretical understanding of the mechanisms and processes involved in these phenomena (Garcia & Hurwitz, 2007; Matias et al., 2020). The traditional person-situation dichotomy is replaced by an emphasis on the mutual impact of the two variables (person and situation) (Funder, 2006). The main idea is that violence involves an influential and continuous interaction between individuals and the various situations they encounter. The *situation* is defined as an actual situation as it is perceived, interpreted, and assigned meaning in the mind of a participant (Magnusson, 1981). Correspondingly, theoretical IPV and IPH perspectives and research should address the situation and proximal events associated with IPV and IPH (e.g., Dixon & Graham-Kevan, 2011; Emery, 2011; Matias, et al., 2020; Vatnar et al., 2017a). These authors have encouraged investigation of “the violence process,” examining the structural and actual context for the violent episode, characteristics of the violent relationship, characteristics of perpetrators and victims, events and conditions preceding an IPV episode, motivations for violent acts, and the outcomes. Applied to IPH, an interactional perspective involves investigating the intimate partner homicide process, by examining the wider set of events and incidents such as (a) sociodemographic and clinical factors, (b) previous IPV, (c) IPH characteristics, and (d) help-seeking that preceded and ended in homicide (Matias et al., 2020; Vatnar et al., 2017a).

### Aim of the Study and Research Questions

The aim of the current study is to track IPHs between 1990 and up to the present in order to investigate developments over time and changes in the following aspects of IPH:

1. Are there any changes in incidence of homicide and IPH in Norway between 1990 and 2020?
2. To what extent have IPH characteristics changed in recent years?
3. Are there any changes in IPH victims’ or perpetrators’ contact with the service providers (including health care and police) in recent years?

## Methods

Since 1990, IPH has been specified as an independent category of homicide—culpable homicide and premeditated murder/with malice as forethought—in the Norwegian Criminal Investigation Service (NCIS) official statistics. NCIS statistics define *homicide* as covering the Norwegian penalty codes for homicides (straffeloven 1902 §233 and straffeloven 2015 §275). IPH is defined as violation of penalty codes for homicide if the perpetrator and victim were current or former intimate partners (currently or previously married or cohabitants). National figures from the Criminal Investigation Service 2020 include homicides and IPHs *without* a final conviction. To investigate changes in homicide and IPH *incidence* over time, including the very last years, these data were applied. Population data were retrieved from Statistics Norway(n d).

The complete cohort of intimate partner homicides in Norway between 1990 and 2019 *with a final conviction* by December 31^st^, 2019 consists of 224 cases, all of which are included in the analyses of *changes in IPH characteristics*. A case with a final conviction includes all cases where a perpetrator was convicted (found guilty) or where the court identified a perpetrator who could not be convicted as, for example, in homicide-suicide cases or cases with insane perpetrators unfit to plead/incompetent to stand trial. *Insane* is defined in the Norwegian penalty code §20: If the perpetrator at the time of the offense was below 15 years old, was mentally disabled (severe), was psychotic, or had severe reduced consciousness. Court case documents were retrieved from the Norwegian Criminal Investigation Service. These court documents contained all material and information collected and used during investigations and court trials. The data were checked for duplicates or missing cases against all cases identified by the Norwegian Criminal Investigation Service.

The study was approved by the Regional Committee for Medical and Health Research Ethics in Norway (2018/1435 REK Sør-Øst B) and by Oslo University Hospital (18-10963_IPHV). The Norwegian Higher Prosecuting Authority provided legal access to the court documents. All cases are included, irrespective of socioeconomic status, race, ethnicity, language, nationality, sex, gender identity, sexual orientation, religion, geography, ability, and age.

### Procedures

Data for the period 1990 to 2012 were collected in 2013 by manually going through the set of court documents for each case and coding the information into quantitative data according to a predefined codebook (Vatnar et al., 2017a) consisting of variables from NCIS homicide statistics and items drawn from three risk assessment tools covering risk factors for IPH: Danger Assessment Revised 20-item (DA-R20) (Campbell & Glass, 2009), Spousal Assault Risk Assessment Guide (SARA) (Kropp & Hart, 2000), and Severe Intimate Violence Partner Risk Prediction Scale (SIVPAS) (Echeburùa, et al., 2009). Data for the period 2013 to 2019 were collected in 2019 using the identical procedure and codebook. The reliability of this procedure was supported by results from an interrater reliability test—intraclass correlation coefficient (ICC), one-way random model, average measures = 0.835, and confidence interval (CI) = [0.714, 0.923]—based on two independent raters’ coding of complete data sets from 20 randomly selected cases (Shrout & Fleiss, 1979). One coder coded all 224 cases. This coder was one of the two coders in the interrater reliability test.

### Measures

Variables and measures of IPH characteristics (e.g., modus operandi and motive) and sociodemographic and contextual factors were drawn from NCIS statistics (See Table 2). Only diagnoses (ICD-10; International Classification of Diseases, 10th Revision) set by health professionals qualified to diagnose mental illness (clinical psychologists and medical doctors) were registered in the variables *perpetrator’s diagnosis* and *victim’s diagnosis*. Perpetrator’s origin was measured as *native citizen, naturalized citizen*, or *foreign citizen*. Perpetrator’s previous conviction was measured *no*, *yes*, or *omit*. The variables (a) perpetrator or victim in contact with police, health, or social service, (b) assessed risk, (c) interventions after assessed risk, and (d) assessed risk communicated to other service providers were measured *no*, *yes*, or *omit*. Categories of IPH were measured as *IPH exclusively, IPH involving multiple victims (all subcategories—for example, children, new partner, and other family members), and IPH and suicide (all subcategories—for example, partner and suicide, partner, children, and suicide*) (see Table 2).

Risk factor items were taken from three validated risk assessment instruments, which include items on IPH. These risk assessment instruments were Danger Assessment Revised 20-item (DA-R20; Campbell et al., 2007; Campbell and Glass, 2009), Spousal Assault Risk Assessment Guide (SARA; Kropp & Hart, 2000; Kropp et al., 1995), and Severe Intimate Violence Partner Risk Prediction Scale (SIVPAS; Echeburùa et al., 2009). Together, these instruments cover a substantial number of possible risk factors of IPH (Vatnar et al., 2017a).

### Analyses

In order to investigate time changes of the homicide and IPH rates, we performed Poisson regression analysis of the 1-year rates with the size of the total Norwegian population for each year as offset. However, the incidences of homicide and IPH were too low for a pattern of the time change to emerge. Thus, data were collapsed into 5 year intervals, and Poisson regression models were conducted to study the time change. Disclosing rate rather than number of incidents in a given time and place is more efficient as it takes into account and controls for possible changes in the total population to provide more accurate measures (Mazerolle et al., 2015). We used Stata version 16 in the statistical analysis.

Univariate and bivariate analyses and multivariate logistic regression analyses were used to test the association between characteristics of IPH and the dependent variable IPH before and after 2012. The stepwise options recommended for logistic regression for small samples were used (Altman, 1991; Pallant, 2010). As suggested by Pallant, initial comparisons of IPH before and after 2012 were carried out by simple descriptive cross-tabulations (Step 1, see Table 2). In the first multivariate logistic regression analyses (Step 2), the interactional theory and findings from the literature were tested within four model categories: (a) sociodemographic and clinical factors, (b) previous IPV, (c) IPH characteristics, and (d) previous help-seeking. Due to the small sample size, the number of variables in each of the models had to be limited. In the forwarded models to the next analytical step, variables with significant (*p* ≤ .05) or trend (*p* ≤ .10) differences between the two dependent variables were included. Significant or trend differences remaining in each of the four model categories in Step 2 were forwarded to Step 3 (Table 3) where they were adjusted for the remaining group differences in Categories a, b, c, and d. Fit of the multivariate logistic regression model was investigated by the Hosmer and Lemeshow test. Values were estimated as model fit indices for the regression models (see Notes in Table 3). Initial analyses and logistic regression analyses were performed using the statistical program package SPSS, version 26.0.

Poisson regression models were conducted to investigate time changes of rates of immigrant perpetrators and victims (Table 4). The standard errors of the incidence rates in the Poisson regression model are estimated in a quasi-Poisson model, and the 95% confidence intervals and the *p*-values are corrected, due to the underdispersion, relative to the Poisson variation.

## Results

### Incidence of IPH

**Figure 1.**
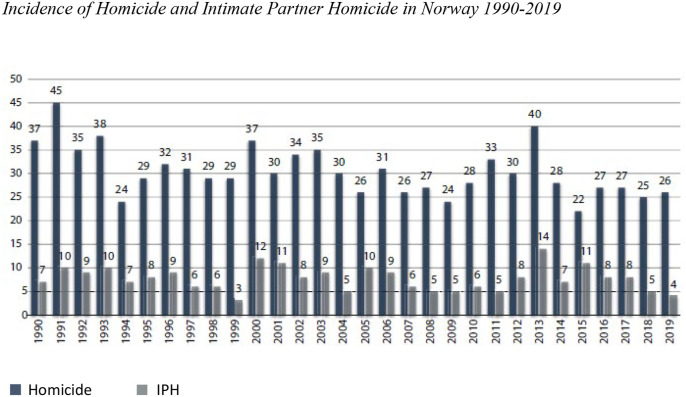
Incidence of homicide and intimate partner homicide in Norway 1990–2019.

Mean annual number of IPH in Norway between 1990 and 2020 was 8, lowest in 1999 (*n* = 3) and highest in 2013 (*n* = 14). For homicide, the incidence rates ratio (IRR) were declining steadily and significantly between 1990 and 2020 (Figure 1 and Table 1). For the last 6 year period, the decline was 41% compared to 1990–1994 (IRR = .592, CI .477–.734, *p* ≤ .001). The IPH incidence rates ratio did not change between 1990 and 2014. In the period 2015–2020, there has been a significant decline in IPH of 35% compared to 1990–1994 (IRR = .645, CI .420–.989, *p* = .044) (Figure 1 and Table 1).

**Table 1.** Incidence Rate Ratio of Homicide and Intimate Partner Homicide in Norway between 1990 and 2020. Poisson Regression.

	IRR (95% CI)	*P*
	Homicide	
Period		≤ 0.001
1990–1994	1	
1995–1999	0.825 (0.663–1.024)	0.082
2000–2004	0.886 (0.717–1.095)	0.957
2005–2009	0.699 (0.551–0.863)	0.001
2010–2014	0.770 (0.622–0.954)	0.017
2015–2020	0.592 (0.477–0.734)	≤ 0.001
	**Intimate partner homicide**	
Period		0.260
1990–1994	1	
1995–1999	0.724 (0.458–1.144)	0.167
2000–2004	0.988 (0.651–1.501)	0.957
2005–2009	0.742 (0.475–1.159)	0.189
2010–2014	0.798 (0.518–1.227)	0.303
2015–2020	0.645 (0.420–.989)	0.044

### Characteristics of IPH

The incidence of IPH-suicide was lower in the last time period (Tables 2 and 3). The incidence of IPH-suicide prior to 2012 was 24.9% and after 2012, 4.3%. This change remained significant after adjusting for other group differences between the periods (Table 3). A smaller percentage of the perpetrators were convicted in Norway previously than in 1990–2012. However, the main reason for this was an increase in the “omit” group—perpetrators who had stayed in Norway for a short time (Table 2 and 3). There has been an increase in sentence length for IPH since the last time period (Table 2 and 3). This change remained significant adjusted for modus operandi, motive, and other IPH characteristics (Table 3).

**Table 2.** Descriptive Data for IPHs 1990–2012 Compared to After 2012 (*N* = 224)

	1990–2012	After 2012	Total	*p*
	(n = 177)	(n = 47)	(N = 224)	
	% ( n )	% ( n )	% ( n )	
Gender (perp)				.292
Male	88.7 (157)	83.0 (39)	87.5 (196)	
Female	11.3 (20)	17.0 (8)	12.5 (28)	
Age (perp.)				.545
Years (mean)	40.1	44.6	41.0	
Marital status				.184
Married	42.4 (75)	57.4 (27)	45.5 (102)	
Cohabitant	32.8 (58)	19.1 (9)	29.9 (67)	
Separated	8.5 (15)	10.6 (5)	8.9 (20)	
Divorced	4.0 (7)	0 (0)	3.1 (7)	
Prev. cohabitant	12.4 (22)	12.8 (6)	12.5 (28)	
Relationship duration				.515
Years (mean)	10.1	13.6	10.8	
Children (perp.)				.336
No	25.7 (45)	17.0 (8)	23.9 (53)	
Mutual to victim	50.3 (88)	61.7 (29)	52.7 (117)	
Previous partner	23.4 (41)	19.1 (9)	25.5 (50)	
Other	0.6 (1)	2.1 (1)	0.9 (2)	
Origin (perp.)				.003
Native Norwegian	66.7 (118)	40.4 (19)	61.2 (137)	
Naturalized citizen	9.6 (17)	21.3 (10)	12.1 (27)	
Foreign citizen	23.7 (42)	38.3 (18)	26.8 (60)	
Origin (victim)				.001
Native Norwegian	72.9 (129)	44.7 (21)	67 (150)	
Naturalized citizen	5.6 (10)	12.8 (6)	7.1 816)	
Foreign citizen	21.5 (38)	40.4 (19)	25.4 (57)	
Education (prep.)				.618
Years mean	10.4	10.3	10.4	
Employment (prep.)				.131
Employed	46.3(82)	34.0 (16)	43.8 (98)	
Not employed	53.7 (95)	66.0 (31)	56.3 (126)	
Previous IPV				.548
No	9.0 (16)	12.8 (6)	9.8 (22)	
1 episode	1.7 (3)	2.1 (1)	1.8 (4)	
2–5 episodes	17.5 (31)	17.0 (8)	17.4 (39)	
> 5 episodes	51.4 (91)	59.6 (28)	53.1 (119)	
Omit	20.4 (36)	8.5 (4)	17.8 (40)	
Previous convicted (perp.)				≤ .001
No	48.0 (85)	31.9 (15)	44.6 (103)	
Yes	45.7 (81)	29.8 (14)	42.4 (92)	
Omit	6.2 (11)	38.3 (18)	12.9 (29)	
Previous convicted (victim)				≤ .001
No	66.1 (117)	40.4 (19)	60.7 (136)	
Yes	17.5 (31)	0 (0)	13.8 (31)	
Omit	16.4 (29)	59.6 (28)	25.4 (57)	
Victim expressed break up				.029
No	27.8 (44)	42.2 (19)	31.0 (63)	
Partly	17.7 (28)	15.6 (7)	17.2 (35)	
Yes	39.9 (63)	42.2 (19)	40.4 (82)	
Omit	14.6 (23)	0 (0)	11.3 (23)	
Custody conflict				.010
No/omit	78.6 (103)	95.5 (42)	82.9 (145)	
Yes/partly	21.4 (28)	4.5 (2)	17.1 (30)	
Mental illness (perp.)				.003
No	23.9 (42)	44.7 (21)	28.3 (63)	
Symptoms	34.1 (60)	12.8 (6)	29.6 (66)	
Diagnosed	36.9 (65)	42.6 (20)	38.1 (85)	
Omit	5.1 (9)	0 (0)	4.0 (9)	
Mental illness (victim)				.025
No	46.0 (81)	70.2 (33)	51.1 (114)	
Symptoms	18.8 (33)	6.4 (3)	16.1(36)	
Diagnosed	25.6 (45)	17.0 (8)	23.8 (53)	
Omit	9.7 (17)	6.4 (3)	9.0 (20)	
Drug misuse (perp.)				.353
No/omit	52.0 (92)	40.4(19)	53.6 (120)	
Yes/partly	48.0 (85)	59.6 (28)	46.4 (104)	
IPH category				.008
IPH (exclusively)	71.2 (126)	91.5 (43)	75.4 (169)	
IPH incl. multiple victims	4.0 (7)	4.3 (2)	4.0 (9)	
IPH incl. suicide	24.9 (44)	4.3 (2)	20.5 (46)	
Motive				.151
Dispute	23.7 (42)	34.0 (16)	25.9 (58)	
Jealousy	40.1 (71)	29.8 (14)	37.9 (85)	
Revenge	6.2 (11)	4.3 (2)	5.8 (13)	
Fear	1.7 (3)	2.1 (1)	1.8 (4)	
Other	11.9 (21)	17.0(8)	12.9 (29)	
Unknown	16.4 (29)	12.8 (6)	15.6 (35)	
Modus operandi				.105
Knife/white weapon	41.2(73)	46.8 (22)	42.4 (95)	
Firearm	24.9 (44)	8.5 (4)	9.8 (22)	
Blunt force	10.2 (18)	8.5 (4)	9.8 (22)	
Strangulation	19.8 (35)	27.7 (13)	21.4 (48)	
Other	4.0 (7)	8.5 (4)	4.9 (11)	
Sentence length (years)				.047
Mean (SD)	10.771(4.484)	14.692 (3.058)	11.841(4.490)	
Contact service providers (perp.)				.096
No	12.6 (22)	10.6 (5)	12.2 (27)	
Yes	78.7 (137)	89.4 (42)	81.0 (179)	
Omit	8.6 (15)	0 (0)	6.8 (15)	
Risk assessment (perp.)				.005
No	45.2 (76)	66.7 (30)	49.8 (106)	
Yes	33.9 (57)	33.3 (15)	33.8 (72)	
Omit	20.8 (35)	0 (0)	16.5 (35)	
Risk intervention (perp.)				.021
No	68.9 (113)	88.9 (40)	73.2 (153)	
Yes	25.0 (41)	13.3 (6)	16.3 (30)	
Omit	6.1 (10)	0 (0)	4.8 (10)	
Risk communicated to other service providers (prep.)				.086
No	74.1 (103)	86.7 (39)	77.2 (142)	
Yes	17.3 (24)	11.1 (5)	14.5 (29)	
Omit	8.6 (12)	0 (0)	4.6 (12)	
Mention IPH to service prov. (perp.)				.001
No	64.7 (108)	91.3 (42)	70.4 (150)	
Yes	8.4 (14)	6.5 (3)	8.0 (17)	
Omit	26.9 (45)	2.2 (1)	21.6 (46)	
Help-seeking private rel. (perp.)				.036
No	27.6 (48)	29.8 (14)	28.1 (62)	
Yes	59.8 (104)	70.2 (33)	62.0 (137)	
Omit	12.6 (22)	0 (0)	10.0 (22)	
Mention IPH to priv. rel. (perp.)				.010
No	51.1 (89)	70.2 (33)	55.2 (122)	
Yes	29.9 (52)	25.5 (12)	29.0 (64)	
Omit	19.0 (33)	4.4 (2)	15.9 (35)	
Contact service providers. (victim)				.001
No	7.4 (13)	19.1 (9)	9.9 (22)	
Yes	72.0 (126)	80.9 (38)	73.9 (164)	
Omit	20.6 (36)	0(0)	16.2 (36)	
Risk assessment (victim)				≤ .001
No	29.1 (50)	56.5 (26)	34.9 (76)	
Yes	40.7 (70)	43.5 (20)	41.3 (90)	
Omit	30.2 (52)	0(0)	23.9 (52)	
Risk intervention (victim)				.023
No	58.4 (97)	76.1 (35)	62.3 (132)	
Yes	30.1(50)	23.9 (11)	28.8 (61)	
Omit	11.4 (19)	0 (0)	9.9 (19)	
Risk communicated to other service providers (victim)				.007
No	67.6 (96)	72.7 (32)	68.8 (128)	
Yes	19.0 (27)	27.3 (12)	21.0 (39)	
Omit	13.4 (19)	0 (0)	10.2 (19)	
Help-seeking private rel. (victim)				.007
No	15.6 (27)	25.5 (12)	17.7 (39)	
Yes	70.5 (122)	72.3 (34)	70.9 (156)	
Omit	13.9 (24)	2.1 (1)	11.4 (25)	
Priv. rel. communicated risk to service providers (victim)				.071
No	73.9 (122)	88.9 (40)	77.1 (162)	
Yes	20.0 (33)	11.1 (5)	18.1 (38)	
Omit	6.1 (10)	0 (0)	4.8 (10)	

*Note:* Mann–Whitney U tests were used for independent groups with non-parametric distribution. Chi square was used for nominal data. Victim age, gender, education, employment, and having children were tested without significant or trend group difference. One person was categorized as *other* for origin of victim. Characteristics for IPV previous to the IPH, categories of IPV, frequencies, severity, etc. were tested without significant group differences or trends. Victim’s drug misuse, victim’s and perpetrator’s suicidal behavior and ideation were tested without significant group difference or trends. IPH motive, Police County, scene of crime, perpetrator and/or victim under the influence of drug or alcohol by the IPH were tested without significant group differences or trends. Modus operandi: *other* = medications, poison, drowning. The following service providers’ relevant factors were tested without significant group differences or trends: Time span between identified risk and the IPH, risk communicated from private relations to perceived providers (perp.), perpetrator mentioned concrete plans of IPH to service providers, victim’s perceived risk for IPH, risk assessment communicated to service providers from private relations (prep.).

**Table 3.** Characteristics for IPH Cases After 2012 Compared to IPH Cases 1990–2012 (Baseline) Multivariate Logistic Regression.

Independent variables	Adj. odds ratio (AOR)	95% CI	*p*
Model 1			
Categories of IPH			.015
IPH (exclusively) (baseline)			
IPH and other vic. all sub cat	1.124	0.211–5.997	891
IPH-suicide all sub cat	0.103	0.022–0.482	.004
Previous convicted (perp.)			≤.001
Yes (baseline)			
No	0.717	0.314–1.636	.412
Omit	9.315	3.405–25.481	≤.001
Origin perp	2.581		.275
Origin vict	5.732		.125
Mental illness perp	7.519		.057
Mental illness vict	5.119		.158
Contact with police, health, and social services (vict.)	3.109		.078
Model 2			
Length of sentence	1.396	1.215–1.603	≤.001
Motive			.021
Dispute (baseline)			
Jealousy	.348	.118–1.025	.056
Revenge	.057	.007–.441	.006
F ear	.908	.857–.062	.908
Other	5.965	.786–45.252	.084
Unknown	.356	.072–1.715	.204
Categories of IPH	3.018		.082
IPH (exclusively) (baseline) modus operandi	2.553		.635
Model 3			
Interventions after assessed risk (perp)	.268	.099–.730	.010
Risk communicated to other services	1.358		.244

*Note* : Multivariate Binary Logistic Regression. Adj. odds ratio (AOR) = Adjusted odds ratio. CI = Confidence Interval.

Model 1: Cox and Snell R Square = .170, Nagelkerke R Square = .264, Hosmer and Lemeshow Test = .254.

Model 2:. Cox and Snell R Square = .253, Nagelkerke R Square = .367, Hosmer and Lemeshow Test = .053.

*Includes only cases where perpetrator was competent to stand trial. Mean value and standard deviation IPH after 2012 = 14.692 (3.058) and 1990–2012 = 10.771(4.484), (*n* = 143).

Model 3: Cox & Snell R Square = .074, Nagelkerke R Square = .110, Hosmer and Lemeshow Test = .999.

*Includes only cases where perpetrator or victim has been in contact with help-seeking/service providers. Perp. = perpetrator (*n* = 184).

The incidence of IPH with previous IPV has not changed. The difference between an incidence of 70.6% compared to 78.7% was not significant (*p* = .548). The incidence rate ratio of immigrants as perpetrators (IRR = .938, CI .328, 2.686, *p* = .905) or as victims (IRR = 1.821, CI .556, 5.967, *p* = .322) of IPH was unaltered when adjusted for population data (Table 4). However, during the period between 1990 and the last year with a complete cohort of final convictions (2017), IRR showed 9 times higher rates for immigrants compared to native Norwegian perpetrators (Table 4) and 7 times higher rates for immigrant victims (Table 4). The incidence for male-perpetrated IPH was unchanged (Table 2). The incidence of perpetrators without a diagnosed mental disorder indicated an increase in the initial analyses (Table 2). However, when adjusted for other sociodemographic differences such as immigration, the difference was not significant (Table 3). Concerning alcohol or drug misuse, there were no changes even in univariate analyses (Table 2).

**Table 4.** Adjusted Incidence Rate Ratio of IPH perpetrator’s and Victim’s Origin Between 1990 and 2017. Poisson Regression.

	Perpetrator’s origin		Victim’s origin	
Variable	IRR (95% CI)	*p*	IRR (95% CI)	*p*
Origin				
Norwegian	1		1	
Immigrant	9.00 (7.18–8.57)	<0.001	6.82 (5.44–8.56)	<0.001
Period		<0.001		<0.001
1990–1994	1		1	
1995–1999	0.703 (0.488–1.129)	0.058	0.710 (0.499–1.010)	0.057
2000–2004	0.861 (0.615–1.206)	0.383	0.882 (0.636–1.222)	0.451
2005–2009	0.570 (0.398–0.816)	0.002	0.591 (0.417–0.838)	0.003
2010–2014	0.503 (0.352–0.721)	<0.001	0.540 (0.382–0.764)	<0.001
2015–2017	0.460 (0.305–0.694)	<0.001	0.480 (0.320–0.719)	<0.001

Note: Model perpetrator - Deviance goodness-of-fit = 3.45827, Prob > chi2(5) = 0.6297, Pearson goodness-of-fit = 3.348525, Prob > chi2(5) = 0.6464. Model victim - Deviance goodness-of-fit = 3.339512, Prob > chi2 (5) = 0.6478 Pearson goodness-of-fit = 3.141786 Prob > chi2(5) = 0.6781. Possible interactions between period and immigration were tested. There was no significant interaction for perpetrators or victims.

### Contact with Service Providers Previous to the IPH

The incidence of IPH with a previous violence risk assessment was unchanged (Table 2). There were also no changes in communicating identified risk to other service providers, including police or health care (Table 2). IPHs perpetrated subsequent to preventive interventions towards the perpetrator were lower after 2012 (Table 2 and 3). This change remained significant when adjusting for other group differences concerning contact with service providers and IPH characteristics (Table 3).

## Discussion

### Main Findings

The IPH incidence rate did not change between 1990 and 2014. From 2015, however, the IPH incidence rate ratio was reduced by 35% compared to the initial period investigated (1990–1994). After 2012, the proportion of IPH-suicide cases and cases with previously crime-involved perpetrators declined. We also found lower rates of IPH perpetrated subsequent to preventive interventions with the perpetrator during this recent period compared to the 1990–2012 period. No changes were observed in the following characteristics: (1) IPH with previous IPV, (2) immigrants as perpetrators and victims, (3) male compared to female-perpetrated IPH, (4) perpetrators diagnosed by mental health diagnosis, (5) alcohol or drug misuse by perpetrators and victims, (6) IPH with a previous violence risk assessment, and (7) communication of identified risk to other service providers, including police or health care. Sentence length for IPH was the only factor having increased after 2012.

### Incidence of IPH

As in the rest of Europe and Canada, Norwegian IPH rates have declined in recent years, albeit at a slower pace than for homicides in general (Caman et al., 2017; Dawson et al., 2009; UNODC, 2019). These patterns contrast with findings from other regions of the world, where intimate partner and family-related homicide is still increasing (UNODC, 2019).

As in our study, most of the literature examining IPH changes over time only cover data back to the 1990s. Prior to that, homicide research did not separate IPH from other types of homicide, although recent research on the topic has recognized the importance of examining IPHs as a separate entity.

### Characteristics of IPH

More than 7 out of 10 IPH cases had a history of IPV (Table 2). This proportion has not changed during the period investigated. The central position of IPV in IPH cases concurs with global findings and has been supported in two recent meta-analyses (Matias et al., 2020; Spencer & Stith, 2018). Killings carried out by intimate partners are rarely spontaneous or random and should be examined as an extreme act on a continuum of intimate partner violence that remains underreported and too often ignored (UNODC, 2019; Vatnar et al., 2017a,b). In a majority of cases, at-risk individuals could be identified and interventions could be employed by multi-agency, coordinated community responses and structured professional risk assessment and management, with considerable preventive potential (Kropp & Hart, 2015; Robinson & Tregidga, 2007).

The proportion of female to male victims of IPH was 7 times higher in Norway. This is higher than in the rest of Europe, where the IPH rate of female victims is 4 times higher than for males (UNODC, 2019). Norway has distinctly low homicide rates (0.5 per 100,000 residents) but the proportion of women victims among all homicides and proportion of women killed in IPH (48%) tends to be higher than in regions with higher levels of homicide. This observation is in line with the first of “Verkko’s laws,” which holds that the higher the level of homicide, the smaller the proportion of female victims and perpetrators (Verkko, 1951). A suggested explanation to the proportional difference is that more women are killed outside the family sphere in countries with higher homicide rates. In other words, the actual IPH and family-related homicide rate may still be high in countries with low proportions of female IPH and female-related homicides (UNODC, 2019). In contrast to prior studies, our study did not find distinct time changes for IPH rates depending on the gender of the perpetrator (Caman et al., 2017; Dawson et al., 2009; Fox & Zawitz, 2007).

Sentence length in IPH cases increased from 2012 onwards, reaching a mean of nearly 15 years in the latter part of the investigated period. This concurs with revisions to the Norwegian penalty codes for homicide in 2009. Norway and most other European countries have not imposed legislative provisions in order to prosecute gender-related killings (femicide) as a separate legal category. Still, our data showed increased sentence length in IPH cases, even without such a separate legal category.

Homicide-suicide is a rare type (4.8%) of homicide, but far more frequent in IPH cases (between 27% and 32%) (Campbell et al., 2007; Vatnar et al., 2019a). In Norway, the proportion of IPH-suicides has considerably decreased in recent years, from 25% in the years before 2012, to 4% in the years after 2012. A similar development has been observed in Sweden (Caman et al., 2017), indicating similar rates for IPH-suicide as for homicide-suicide in general. The likelihood of committing suicide after perpetrating IPH is more than 8 times higher than for other homicides (Matias et al., 2020). In the U.S., almost half of IPH-suicides were carried out with a firearm (Smucker et al., 2018). A suggested explanation for the low incidence of IPH-suicide in Norway and Sweden might be that few IPHs in these countries are carried out with a firearm.

Immigrants were overrepresented among victims and perpetrators of IPH in our study. This is supported by a recent meta-analysis concluding that belonging to an ethnic minority is a common characteristic for both the victims and perpetrators (Matias et al., 2020). However, the overrepresentation of immigrants was stable throughout the period investigated, in contrast to public discourses claiming increased incidence of IPH among immigrants. Cultural competency is also an aspect to consider regarding risk assessment (Singh et al., 2011). Immigrant status seems to have little to do with ethnicity per se, but could rather be interpreted as a contextual risk factor indicating barriers related to the migration process, immigrant status, social exclusion, and economic, personal, or social dependence (Gonçalves & Matos, 2016; Vatnar et al., 2017b). In addition, de Soysa and Noel (2020) analyzed global homicide rates in more than 40 countries between 1995 and 2013. They found that ethnic *heterogeneity* had an inverted U-shaped association with homicide rates and that ethnic polarization and ethnic dominance rather than diversity were associated with homicide rates. Moreover, it should be noted that most studies related to ethnicity and IPH have been carried out in the U.S., whose multicultural diversity differs from Europe’s (Matias et al., 2020). In accordance with this, our study from Norway contributes to an analysis of these factors within a European context.

The perpetrator-related risk factor—previously convicted—was hard to compare because a substantial proportion of perpetrators had stayed in Norway for a short time. Due to this, we had a shorter time frame for historical data and no documentation of previous criminal convictions from abroad. Therefore, risk assessment might be even more problematic concerning immigrants with short stays in the actual context. It is challenging to communicate the fact that IPH is distributed in a socially biased manner, implying that population groups already characterized by accumulated welfare deficiencies are at increased risk for IPH. Rather than interpreting this in a stigmatizing manner, it is essential to use this information proactively to identify preventive efforts towards groups where the potential can be substantial. Explicitly, risk assessment should consider interactions of components of situations, structures, and persons, not dichotomizing or simplifying risk factors. Equal to the risk of labeling immigrants is the risk of stigmatizing perpetrators or victims with a mental disorder or alcohol or drug misuse.

### Contact with Service Providers Previous to the IPH

Our findings that IPHs perpetrated subsequent to preventive interventions towards the perpetrator were lower after 2012 concurs with findings in a recent meta-analysis, showing that perpetrator risk factors increased more strongly the odds of an IPH occurring, compared to victim’s risk factors. This suggests that it may be more important to do risk assessment and interventions using factors related to the perpetrator than to the victim when assessing the potential of an IPH occurrence (Spencer & Stith, 2018). One third of the IPH cases involved violence risk assessment prior to the homicide. The proportion did not change over time, although, in 2013, the Norwegian police were formally instructed by the National Police Directorate to conduct structured risk assessment by using SARA in every case of IPV.

Structured risk assessment requires resources. However, monetary resources are insufficient if they are not accompanied by strengthening professional expertise within the field. Furthermore, it is crucial to strengthen liaisons between and within the systems. We found no changes in recent years in the communication of identified risk to other service providers, including police or health care. Evidence-based risk factors for IPH highlight the importance of service providers (including police and health care) acknowledging the seriousness of the risk factors and not only being aware of, but also communicating the risk to IPV perpetrators and victims, as well as to other service providers to inform preventive measures (Spencer & Stith, 2018).

### Study Limitations

The high-quality databases on homicide and population data in Norway—NCIS and National Statistics Norway—are unique sources of information. They also include offenders who committed suicide in commission of an offense and perpetrators later deemed unfit to stand trial due to mental illness. The lack of information on IPH-suicide has been a deficiency in the research literature (Caman et al., 2017; Kivisto, 2015). Almost every (99%) homicide in Norway has a known perpetrator-victim relationship. This is a major advantage in terms of representativeness. When using data spanning three decades, it is important to acknowledge that reporting and recording may change over time and confound any observed changes, in particular, those regarding convictions and case characteristics. One example is the reduced incidence of omitted/missing values for variables registered in the cases after 2012. This demonstrates the fact that criminal records have grown considerably in scope/size, as illustrated by the fact that the 47 cases after 2012 amounted to 79,384 pages. Even though the Norwegian sample consists of the entire cohort of IPH in Norway between 1990 and 2019 with a final conviction, the sample is small. Although the time range is wide, our Poisson regression analyses and multivariate logistic regression analyses are robust. However, there might be changes and group differences showing non-significant results due to the small sample size and small subgroups representing a risk for statistical Type II errors.

The variables used in the present analysis did not cover all possible risk factors of IPHs. Criminal case documents relating to each of the 224 IPHs were the only data source in this study. These documents are produced for purposes other than research and consequently did not provide exhaustive data related to our research questions. Accordingly, there may be a risk of false negatives—for example, failure to identify diagnoses of mental illness, previous IPV, and help-seeking.

A caveat in the current study of risk factors is that we have not used comparison samples, as, for example, non-fatal IPV. In addition, the elevated percentage of unknown criminal history due to short stays in Norway is a limitation for the analysis of this risk factor. Overall, even if we found results similar to other IPH studies, our study is predominantly an investigation of the Norwegian time changes in IPH. Except for the sentence length for homicide (incl. IPH), which was changed in 2009, to our knowledge there have not been other changes that may have affected the changes presented in this paper.

### Implications for Practice, Policy, and Research

Meta-analyses (Matias et al., 2020; Spencer & Stith, 2018) support risk factors in recognized risk assessment tools like SARA (Kropp et al., 1995) and The Danger Assessment (Campbell & Glass, 2009), both of which were used in our study. Matias et al. advocate that risk assessment must take into account that there are types of IPV with greater predictive power of lethality. However, due to the low base rates of IPH, the predictive power of lethality is low. In accordance with these nuances, it is important that service providers do not stereotype risk profiles for victims and perpetrators in the absence of empirical evidence (Spencer & Stith, 2018). It is still critically important that service providers (including police and health care) incorporate their own professional judgment when conducting risk assessments (Kropp & Cook, 2013). Further research is needed on the difference and nuances between IPH and non-fatal IPV, on IPH compared to other categories of homicide, subgroups of IPH (IPH-suicide, female perpetrators, etc.), and IPH risk factors like history of violence, criminal record, and mental disorders (Matias et al., 2020). The fact that there was no change in the incidence of IPH cases in which identified risks were communicated to other parts of the system, indicates that awareness, attitudes, and behaviors concerning duty of confidentiality, duty to provide information, and mandatory reporting are still scarce (Vatnar et al., 2019b).

## Conclusion

IPH is often the culmination of long-term violence and can be prevented (Matias et al., 2020; Spencer & Stith, 2018). The complexity of the most severe IPV cases indicates that victims and perpetrators need access to a comprehensive range of services provided by the police and justice system and by the health and social services. To be effective for population groups already characterized by accumulated welfare deficiencies, interventions must be coordinated and customized for at-risk groups. It is important that strategies and services aimed at combating IPV include provisions dealing with extreme forms of IPV such as IPH (UNODC, 2019).
